# Comparison of quantitative lung measures in low dose energy-integrating detector and photon-counting detector chest CT with an anthropomorphic phantom

**DOI:** 10.1088/2057-1976/ae0e27

**Published:** 2025-10-30

**Authors:** Natally AlArab, Marrissa J McIntosh, Junfeng Guo, Jarron Atha, Abhilash S Kizhakke Puliyakote, Jessica C Sieren, Eric Hoffman, Ehsan Abadi, Sean B Fain

**Affiliations:** 1 Department of Radiology, Carver College of Medicine, University of Iowa, Iowa City, IA, United States of America; 2 Roy J Carver Department of Biomedical Engineering, University of Iowa, Iowa City IA, United States of America; 3 Department of Radiology, Duke University, Durham NC, United States of America

**Keywords:** PCD-CT, kernels, lung, densities, comparing, airways, low dose

## Abstract

Photon-counting detector (PCD) computed tomography (CT) promises improved resolution and contrast at reduced x-ray dose compared to energy-integrating detector (EID) CT. Our objective is to determine the parameters that achieve robust accuracy of quantitative measures in chest PCD-CT studies compared to quantitative EID-CT at low CT dose. The Kyoto LUNGMAN chest phantom with preserved lung tissue core and NIST-calibrated foam density standards (4–20 lbs.), and the COPD Lung Phantom II with six airways of various outer and inner diameters, were scanned using PCD-CT (NAEOTOM Alpha) and EID-CT (SOMATOM Force) with a target CT dose index (CTDI_vol_) of 2.2 mGy to match that specified for ongoing longitudinal quantitative chest CT studies of chronic lung disease. Mean density of foam inserts in the Kyoto phantom and mean lumen area (LA) and wall thickness (WT) of COPD Lung Phantom II were automatically segmented, analyzed, and compared using the root mean squared error (RMSE). Contrast-to-noise (CNR) and signal-to-noise (SNR) ratios were also automatically calculated. Large (11.2 mm) and small (5.5 mm) airway LA and WT in the lung tissue core of the Kyoto phantom were semi-automatically measured. PCD-CT with the Qr40 kernel yielded superior foam density accuracy (RMSE: 6.1–7.8 HU) compared to EID-CT (RMSE: 9.7 HU). Q+UHR mode with Qr64 and a 1024 × 1024 matrix achieved the highest airway accuracy (RMSE <1.8 mm^2^ for LA and <0.3 mm for WT). However, these protocols showed increased variability in tracheal air measurements (SD up to ±9 HU), indicating a trade-off between higher spatial resolution and measurement repeatability. At equivalent low radiation dose (2.2 mGy CTDI_vol_), PCD-CT outperforms EID-CT in quantitative accuracy for foam density and airway measurements, with comparable SNR and CNR. These results support the use of PCD-CT for quantitative lung imaging in longitudinal studies, provided reconstruction settings are selected to balance accuracy and repeatability.

## 1. Introduction

X-ray computed tomography (CT) imaging has become a mainstay technology for the visual assessment of abnormalities in the pulmonary parenchyma, airways, and vasculature, to diagnose and monitor the progression of chronic lung disease (e.g., emphysema, fibrosis), and identify the clinical phenotypes [1–3]. The small airways (<2 mm in diameter) represent a ‘silent zone’ of the lung where disease may progress extensively without being detected using conventional pulmonary function tests [4]. However, the delineation and measurement of these submillimeter structures are limited using conventional energy integrating detector (EID) CT due to detector design (e.g., element size, optically isolating septa) and electronic readout noise. Photon-counting detector CT substantially overcomes these limitations [5, 6] and can achieve increased resolution compared to EID-CT, at similar or lower radiation doses.

An initial clinical investigation of older healthy volunteers demonstrated that images of the lung obtained using low-dose PCD-CT had better diagnostic quality, including increased image quality for assessment of lung tissue and lung nodules, lower subjective image noise and less pronounced beam hardening, than those obtained via EID-CT [7]. Another investigation scanned clinical patients who had previously undergone low-dose EID-CT and showed improved signal-to-noise ratio (SNR), better image quality and increased sharpness compared to EID-CT [8], with reduced radiation dose. Finally, both PCD-CT and EID-CT images were acquired in patients suspected of interstitial lung disease (ILD) [9], which were subsequently scored by radiologists for image quality and confidence in the presence or absence of abnormal imaging findings. Radiologists were more confident in imaging findings for reticulation, ground-glass opacities, and mosaic attenuation on PCD-CT than EID-CT. Together, these findings show the feasibility of using low-dose PCD-CT for the qualitative assessment of pulmonary abnormalities in the clinic.

Recent studies have extended these findings into the quantitative imaging domain. For example, Kerber *et al* [10]. demonstrated that PCD-CT enables accurate low-dose emphysema quantification using automated scoring algorithms, showing strong correlation with visual scoring metrics. Similarly, Fletcher *et al* [11]. Reviewed the early integration of PCD-CT into thoracic imaging practice, highlighting its superior performance for quantitative assessment of airway wall thickness, emphysema burden, and lung parenchymal density. These findings support the growing role of PCD-CT in clinical quantitative imaging, beyond visual diagnostics.

Previous phantom studies have investigated PCD-CT image quality at reduced radiation doses [12] and finer spatial resolution [13]. However, there has not yet been a study that comprehensively evaluates the impact of acquisition and reconstruction protocols on quantitative analyses of lung density and airway morphometry typically used for longitudinal studies of obstructive lung disease. In particular, multiple ongoing multi-center studies are using low-dose quantitative chest CT with EID-CT systems to evaluate air trapping, emphysema and bronchiectasis in longitudinal studies of COPD and asthma [14–16]. There is a paucity of evidence that PCD-CT can be used to obtain comparable or superior measures of these pathologies using equivalent low-dose protocols. The objective of this study was to use chest phantoms with reference standards of known values to preclinically establish low-dose PCD-CT acquisition and reconstruction protocols for quantitative chest imaging. Some of these findings have been previously published in the form of two abstracts [ 17, 18].

## 2. Materials and methods

### 2.1. Study protocol

Two phantoms were scanned three times each using EID-CT (SOMATOM Force, Siemens Healthineers) and PCD-CT (NAEOTOM Alpha, Siemens Healthineers) using parameters as described in table 1. The EID-CT parameters [19–21] were selected to match that used for ongoing longitudinal trials of chronic lung disease and some PCD-CT parameters were matched to be as close as possible, while others were varied, intending to take advantage of improved noise performance and spatial resolution. Parameters that were matched to the extent possible included target CT dose index (CTDI_vol_), 2.24 mGy (EID-CT) versus 2.22 mGy (Quantum+, Q+) and 2.27 mGy (Quantum+ultra-high resolution, Q+UHR); slice thickness 0.75 mm (EID-CT) and 0.8 mm (Q+ and Q+UHR), tube voltage of 120 kV, and pitch of 1.0 in all cases. Architectural characteristics and desire for enhancing resolution led to differences, including collimation 192×0.6 mm (EID-CT); 144 × 0.4 (Q+) and 120 × 0.2 (Q+UHR); and tube rotation time of 0.5 s (EID-CT) versus 0.25 s (Q+ and Q+UHR). To explore spatial resolution performance for PCD-CT, reconstruction with both Qr40 versus Qr64 kernel configurations were included. Quantum Iterative Reconstruction (QIR) levels 2 and 4 were applied in conjunction with Qr40 and Qr64 kernels, respectively, to reflect commonly used clinical settings across 512 × 512 and 1024 × 1024 matrix sizes. QIR2 was selected to balance noise suppression and structural detail in standard-resolution imaging, while QIR4 was used to offset the increased noise associated with the sharper Qr64 kernel in high-resolution protocols.

### 2.2. Structure and configuration of chest CT phantoms

**Figure 1.**
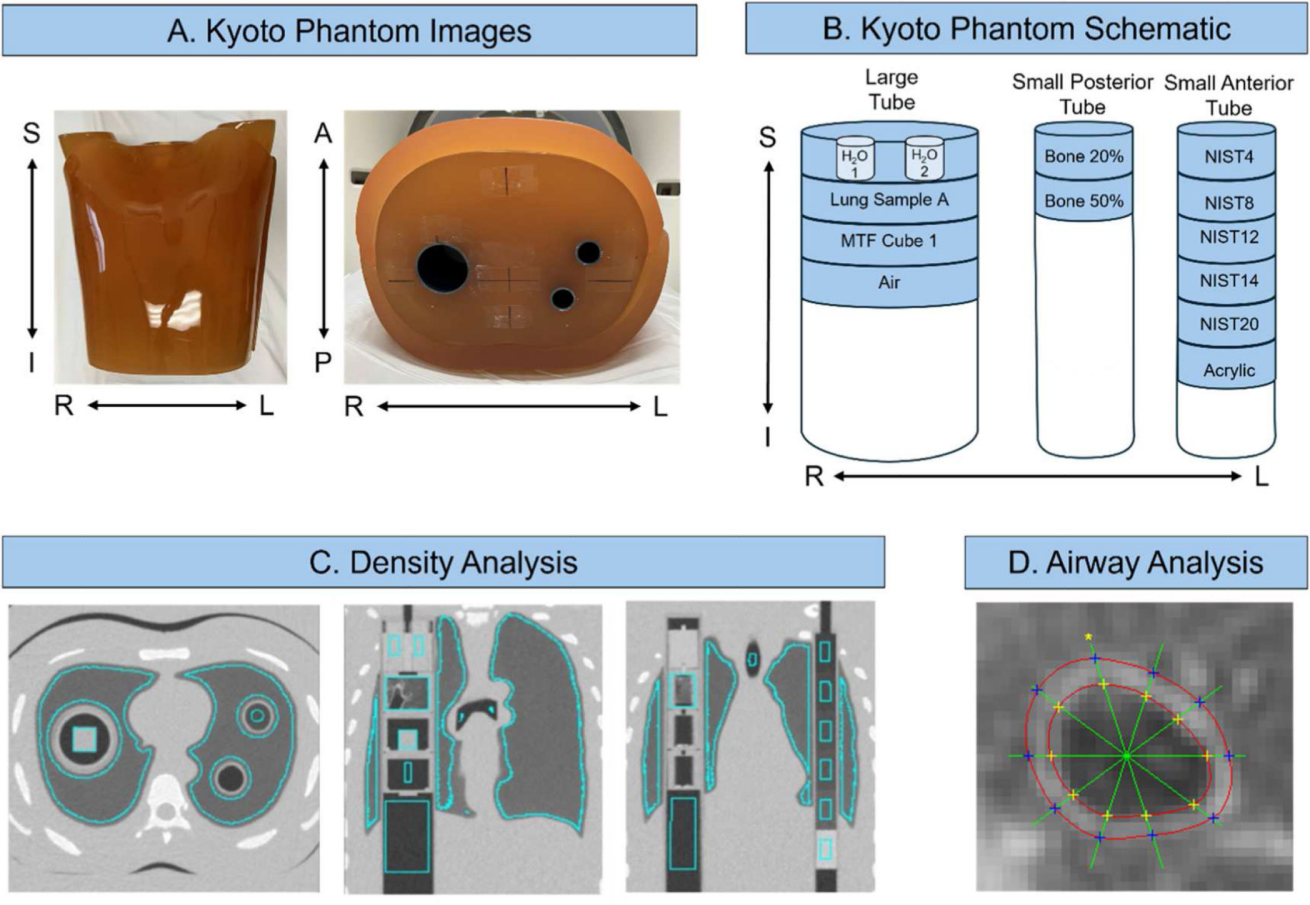
Kyoto phantom schematic and analysis. (A) Image and cross section of anthropomorphic chest phantom (Kyoto ‘LUNGMAN’) with arrows showing Superior (S), Inferior (I), Right (R), Left (L), Anterior (A) and Posterior (P) directions. (B) Schematic of tube inserts in Kyoto phantom with arrows showing Superior (S), Inferior (I), Right (R) and Left (L) directions. (C) CT scans (axial and coronal views) of Kyoto phantom showing the ROI (blue) generated from the automated density analysis on Pulmonary Analysis Software Suite. (D) Semi-automated analysis of Kyoto Phantom Lung Sample A airway analysis.

Two CT phantoms were used in this study: an anthropomorphic chest phantom and a reference standard ‘Test Object.’ The modified anthropomorphic chest phantom [22] (Multipurpose Chest Phantom N1 ‘LUNGMAN’; Kyoto Kagaku Co. Ltd, Kyoto Japan), shown in figure 1(A), was used to assess densitometry across all acquisitions; details about phantom construction have been previously published [23]. In summary, this phantom is comprised of models for the lungs, ribs, spine, sternum and scapulae made of acrylic, polyurethane and epoxy resin and surrounded by detachable chest plates attached to simulate a typical adult body mass index (BMI). The modifications to the Kyoto phantom configuration are detailed in figure 1(B). Three vertically spanning tubes were placed inside the phantom. The right tube contained water samples, lung sample A (preserved pig lung samples from an inflated and over-inflated pair of lungs[24]), a cube (‘MTF Cube 1’ used to calculate the modulation transfer function ) and air (air inside lung or lung air). The small posterior tube contained 20% and 50% bone equivalent density standards, respectively, and the small anterior tube contained five stacked foam reference standards (4,8,12,14 and 20 lb./ft), three of which (4, 12, and 20 lb./ft) were qualified by the National Institute of Standards and Technology (NIST) [3, 25], yielding a known reference density (table 2), and finally an acrylic standard. True density measurements for each material are shown in table 2.

**Figure 2.**
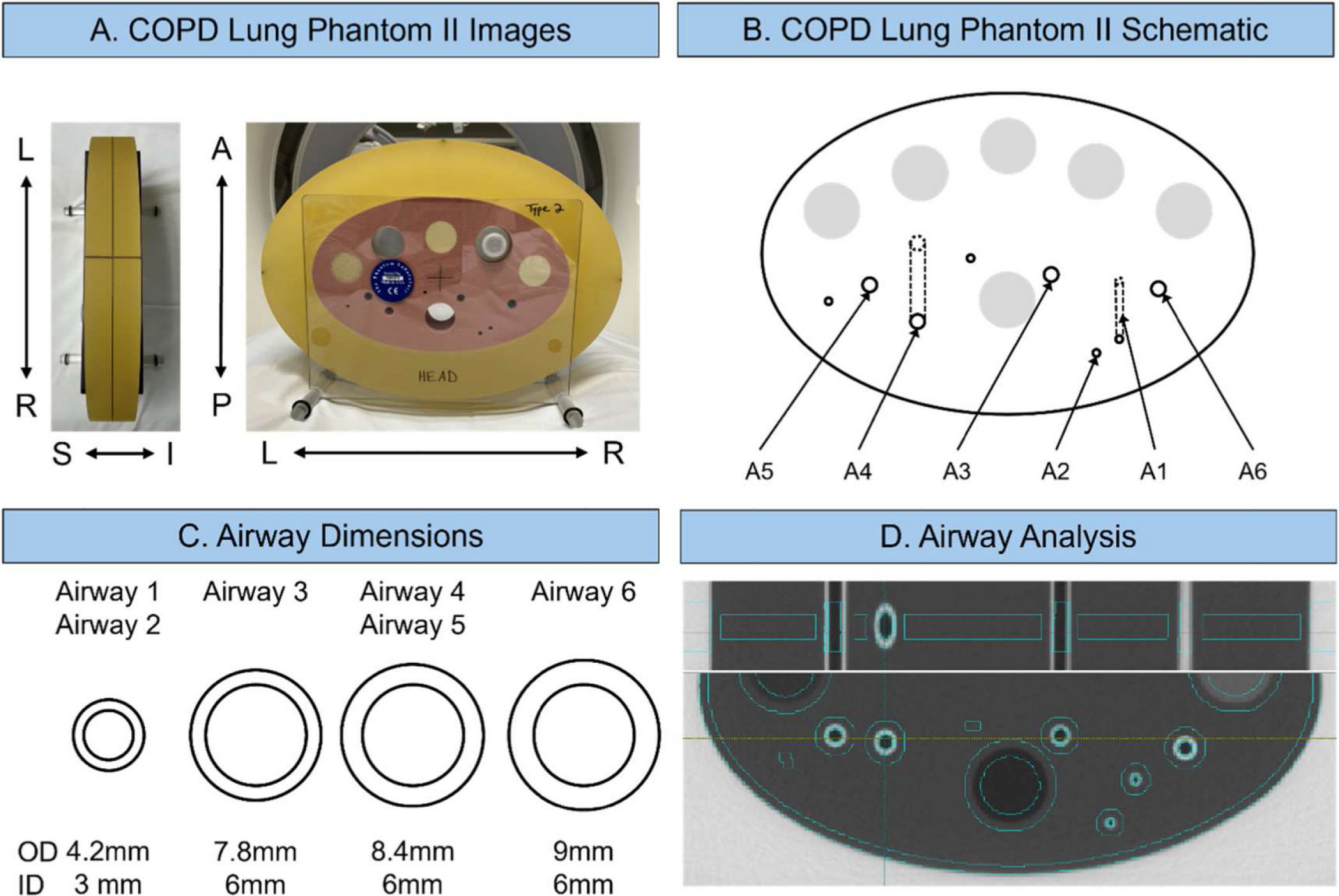
COPD lung phantom and analysis. (A) Image and cross section of COPD Lung Phantom IICCT162 with arrows showing Superior (S), Inferior (I), Right (R), Left (L), Anterior (A) and Posterior (P) directions. (B) Schematic of airway (A) tube placement within COPD Lung Phantom II. Dashed lines indicate airways (A1 and A4) that are placed within the phantom at 30° angle from scanner *z* -axis. (C) Schematic of airway tube dimensions with outer diameter (OD) and inner diameter (ID) below. Airways 1 and 2, and Airway 4 and 5, have the same dimensions, with Airway 1 and 4 at 30° angle from scanner *z*-axis. (D) Automated analysis of COPD Lung Phantom airway tubes.

The second phantom was a CT test object (COPD Lung Phantom IICCT162, Phantom Lab) [26], shown in figure 2(A). This phantom was used to assess airway geometry across all acquisitions. The COPD Lung Phantom II configuration is detailed in figure 2(B) and consists of outer and inner rings with water-like and parenchyma-like attenuation characteristics, respectively. The inner ring contained six embedded polycarbonate tubes with known diameters (table 2 and figure 2(C)) as well as acrylic, water, and NIST 4, 12 and 20 inserts, and an air hole.

### 2.3. Quantitative image analysis

All images were analyzed using in-house developed software - the Pulmonary Analysis Software Suite (PASS) (University of Iowa, Iowa City, Iowa). The analysis of the Kyoto phantom and COPDGene phantom are conducted separately by different modules.

For the customized Kyoto phantom, three tubes are placed inside the body to hold various inserts—one large tube on the right side and two smaller tubes on the left side (figure 1). The ends of each tube will remain empty, allowing for easy identification using a thresholding method. The size and location of the tubes were measured and verified to ensure the correct phantom is used and oriented properly. Based on the known configuration of the inserts, each insert was separated and processed individually according to its type (figure 1(C)).

A customized insert containing a cubic device made of solid dense material was used to measure the Modulation Transfer Function (MTF) along the three axes. Each edge of the cube is 2 cm in length. One of the six smooth, flat sides is glued to the end plate of the empty cylinder insert, while the other five sides remain exposed to air. From these five floating sides, three were selected to measure the MTF along three perpendicular directions, each parallel to one of the axes, using the derivative of the edge response function as previously described [27–29]. For each selected side, multiple rays were cast along the perpendicular direction of the side, and an edge spread function (ESF) was created for each ray. The ESFs of all rays were then averaged to form the ESF for that side. The ESF was differentiated to obtain the line-spread function (LSF), which was subsequently multiplied by a Hann window to reduce noise in the tails. The fast Fourier transform (FFT) of the LSF yields the MTF along the perpendicular axis to the side. In this study, we focused on the in-plane MTF, so the MTFs along the x- and y-axes were averaged to report the in-plane MTF. This study presents the mean in-plane MTF across three repeated scans of the same protocol to evaluate the in-plane MTF.

Airway measurements were performed semi-automatically using PASS (figure 1(D)). For the preserved cores in the Kyoto phantom, a central point was manually placed in one slice of each selected airway (large: ~11 mm diameter; small: ~5.5 mm diameter), and radial rays were automatically generated. The inner and outer boundaries were identified using the Full Width at Half Maximum (FWHM) method, with manual editing permitted for misclassified rays. Although a radiologist did not review segmentation, all measurements were performed by a trained imaging researcher following standardized protocols. The same operator analyzed all datasets to reduce inter-operator variability.

The contrast-to-noise (CNR) and signal-to-noise (SNR) ratios were calculated as follows: figure 1(D) denoting the boundary of the inner and outer radius; rays with ‘+’ incorrectly placed inside or outside the radius (not shown) could be manually edited or excluded. All measurements were performed by a trained imaging researcher following standardized

$$\mathrm{CNR}\;=\;\frac{\left(\mathrm{mean}\;\mathrm{density}\;\mathrm{of}\;\mathrm{NIST}12\mathrm{in}\;\mathrm{image}\;\#1\;-\;\mathrm{mean}\;\mathrm{density}\;\mathrm{of}\;\mathrm{NIST}4\mathrm{in}\;\mathrm{image}\;\#1\right)}{△\mathrm{SD}\;\mathrm{of}\;\mathrm{lung}\;\mathrm{equivalent}\;\mathrm{foam}}*\sqrt{2}$$

$$\mathrm{SNR}\;=\;\frac{\left|\left(\mathrm{mean}\;\mathrm{density}\;\mathrm{of}\;\mathrm{lung}\;\mathrm{equivalent}\;\mathrm{foam}\;\mathrm{in}\;\mathrm{image}\;\#1\right)\right|}{△\mathrm{SD}\;\mathrm{of}\;\mathrm{lung}\;\mathrm{equivalent}\;\mathrm{foam}}*\sqrt{2}$$

Where Δ denotes that SD is calculated from the difference image of the two scans performed using the same protocol.

The dimensions of two airways selected from the preserved cores in the fixed Lung Sample A (large: ~11 mm in diameter, small: ~5.5 mm in diameter) in the Kyoto phantom were semi-automatically measured using PASS (figure 1(D)) with manual editing permitted for misclassified rays. A point was manually placed in one slice at the center of the airway, and a set of rays were automatically generated, with ‘+’ in protocols. The same operator analyzed all datasets to reduce inter-operator variability.

The lumen radius and wall thickness (WT) were also determined using the Full Width at Half Maximum (FWHM) and the lumen area (LA) was calculated as

$$\mathrm{LA}\;=\;\pi {\left(\mathrm{lumen}\;\mathrm{radius}\right)}^{2}$$

For the COPDGene phantom, only the results related to airway measurements are presented here. The dimensions of the airway tubes embedded in the COPD Lung Phantom II were calculated as described previously [27]. In brief, a set of rays is automatically defined at the center of each tube, as shown in figure 2(D). The FWHM method is then applied to identify the boundaries of the airways, from which the lumen radius and wall thickness are determined. The LA of each tube is subsequently calculated as described earlier.

### 2.4. Statistical analysis

Root means squared error (RMSE) was used to compare measured densities and airway LA and WT across protocols to known reference values, shown in table 2. The standard deviation (SD) across repeated scans was also evaluated as a measure of interscan repeatability.

## 3. Results

### 3.1. Kyoto phantom density

**Figure 3.**
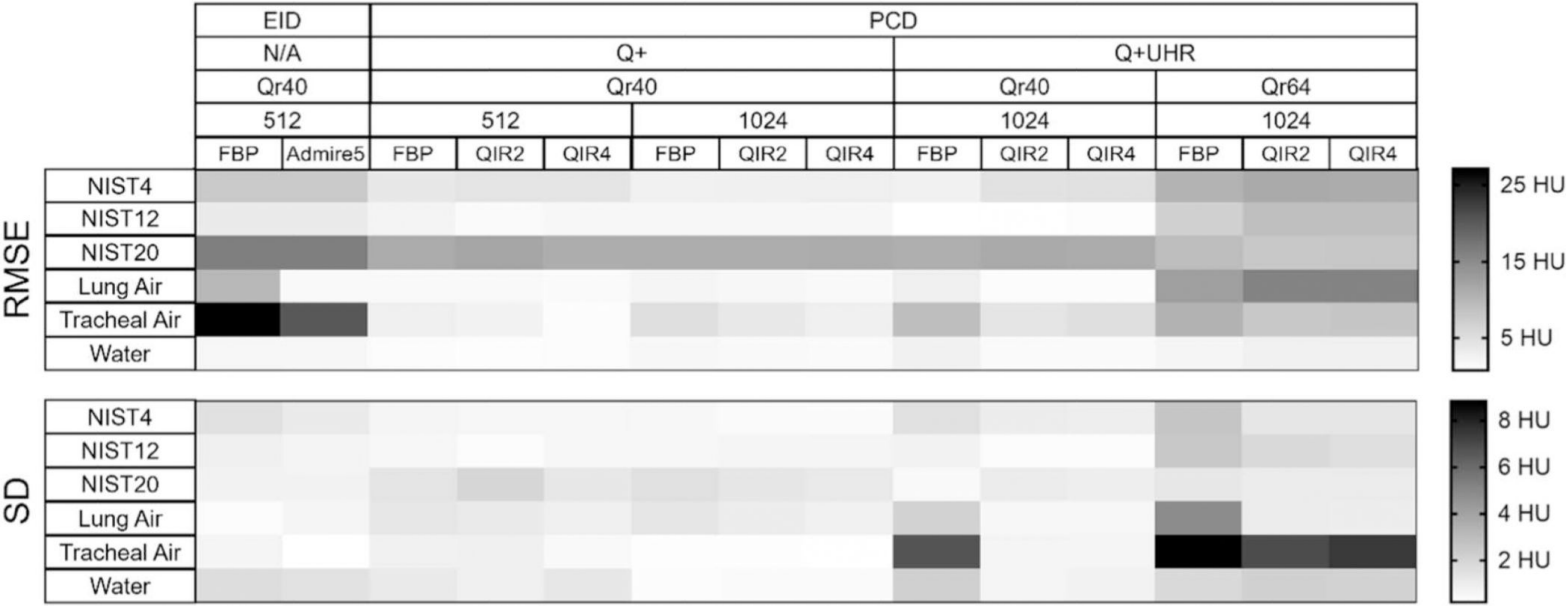
Kyoto phantom density analysis Heatmap representations showing RMSE between mean measured and true density across protocols (top) and SD of mean density across three repeated scans (bottom). Lower RMSE values (lighter color) indicate better agreement with reference standards. PCD-CT protocols using Qr40 consistently outperformed EID-CT across most foam densities. Top: RMSE of 3 repeated scans for EID and PCD protocols for Kyoto Phantom Density foams show relatively consistent performance across reconstruction techniques for PCD protocols. For NIST4, NIST12, and Air foams, PCD Q+UHR Qr64 1024 matrix protocols resulted in mean density further from true density than EID protocols; all other PCD protocols outperformed EID for these foams. For NIST 20 and Tracheal Air, all PCD protocols outperformed EID protocols for mean density measurement. PCD and EID protocols were similar at measuring density of water. Bottom: The standard deviation was within approximately 2 HU for three repeated scans for all measurements and all protocols, except using PCD Q+UHR Qr64 1024 matrix with FBP reconstruction for NIST4, NIST12, Air and Tracheal Air. True Densities: NIST4=−939 HU, NIST12=−822 HU, NIST20=−697HU, Air=−1000 HU, Tracheal Air=−1000 HU, Water=0 HU.

Figure 3 shows heat maps corresponding to RMSE values for Kyoto Phantom Density measurements across scanners and protocols. The RMSE for densities measured in PCD-CT protocols that used Qr40 reconstruction were lower than RMSE for EID-CT protocols. The RMSE for water, however, was relatively similar across all protocols, including Q+ and Q+UHR, and scanners. PCD-CT protocols that used Qr64 reconstruction had greater or similar RMSE as compared to EID-CT protocols. For the Qr64 kernel reconstruction with PCD-CT protocols using Q +UHR mode and 1024 × 1024 matrix, the RMSE was on the order of 15 HU, substantially higher compared to both EID-CT protocols. Furthermore, the density of tracheal air (−1000 HU) in PCD-CT reconstructions was less accurate across protocols, with Q+UHR mode and the Qr64 kernel showing higher RMSE compared to other PCD-CT protocols, with differences of up to 20 HU. Nevertheless, all PCD-CT protocols significantly outperformed or were similar to EID-CT, which had an RMSE on the order of 24 HU (table S2).

For NIST4 (−939 HU) and NIST12 (−822 HU), the EID-CT protocol performed well with RMSE for density measurements of less than 5 HU. Equivalent, to slightly improved, accuracy of the NIST4 and NIST12 density measures, RMSE within 0–2 HU, were obtained for the different PCD-CT protocols using Q+ and Q+UHR mode with the Qr40 kernel and both the 512 × 512 and 1024 × 1024 matrices. By comparison, PCD-CT protocols using Q+UHR mode with the Qr64 kernel and a 1024 × 1024 matrix demonstrated the least accurate density measures with the highest RMSE values on the order of 11 HU.

The accuracy for the higher density NIST20 (−681 HU) foam was lower for all protocols with RMSE ranging from 8–16 HU. EID-CT reconstructions displayed the highest RMSE values showing inferior performance to PCD-CT for all protocols. PCD-CT protocols using Q+UHR mode with the Qr64 kernel and 1024 × 1024 matrix demonstrated slightly better density accuracy compared to the other PCD-CT protocols with an RMSE difference of less than 4 HU.

Water densities (0 HU) exhibited negligible RMSE differences across scanners, demonstrating robust accuracy irrespective of the protocols used for PCD-CT and EID-CT.

All density measures for PCD-CT were similar for all iterative reconstructions, except for tracheal air which had decreasing RMSE across FBP, QIR2 and QIR4 for less than 3HU.

The bottom of figure 3 also shows that PCD-CT protocols, particularly those using the Q+UHR mode with Qr64 kernel and 1024 × 1024 matrix, demonstrated higher variability of the mean density measures across repeated scans compared to EID-CT. High reproducibility was still observed for PCD-CT, however, for NIST standards (NIST4, NIST12, and NIST20) and water; SD values remained within 2 HU for most PCD-CT protocols. However, for air densities, especially tracheal air, variability was more pronounced in PCD-CT protocols, mainly those using Q+UHR mode with the Qr64 kernel but also for the Qr40 kernel with FBP reconstruction, where SD values increased by more than 8 HU. Despite these variations, PCD-CT protocols still displayed comparable consistency compared to EID-CT across density standards for QIR 2, and 4 iterative reconstructions.

Overall, PCD-CT protocols, especially those using the Qr40 kernel, demonstrated superior or comparable accuracy and reproducibility in lung-mimicking foam density measurements compared to EID-CT, except for tracheal air, where higher variability was observed in Q+UHR high-resolution reconstructions.

### 3.2. COPD lung phantom II airways

**Figure 4.**
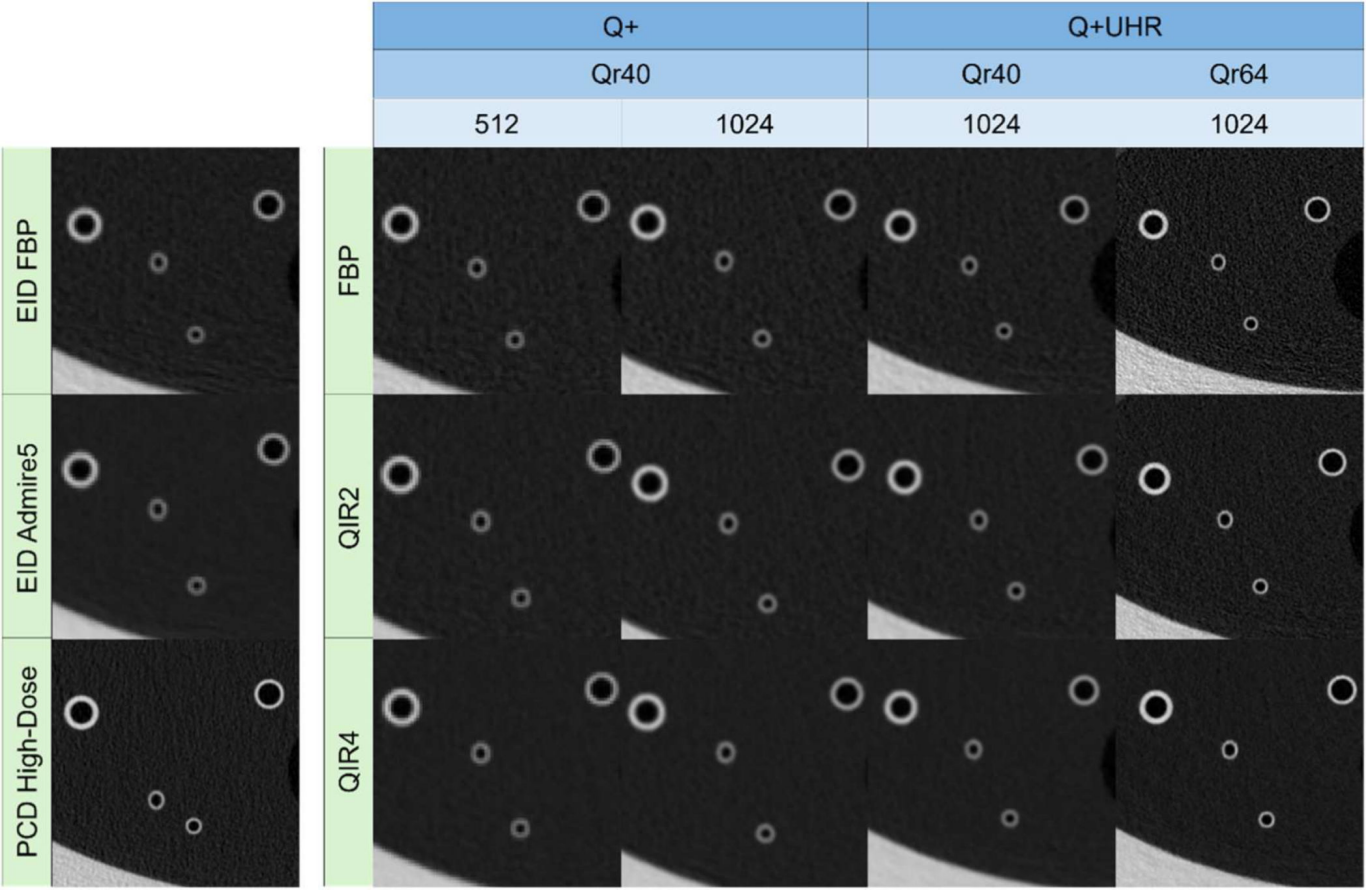
Representative images from COPD lung phantom II for each protocol. Qualitative comparison of COPD Lung Phantom II images for EID FBP, EID Admire5 and PCD high-dose (left) and each low-dose PCD protocol (right). Airway tubes shown for airway 6 (top left), airway 3 (top right), airway 1 (middle) and airway 2 (bottom).

**Figure 5.**
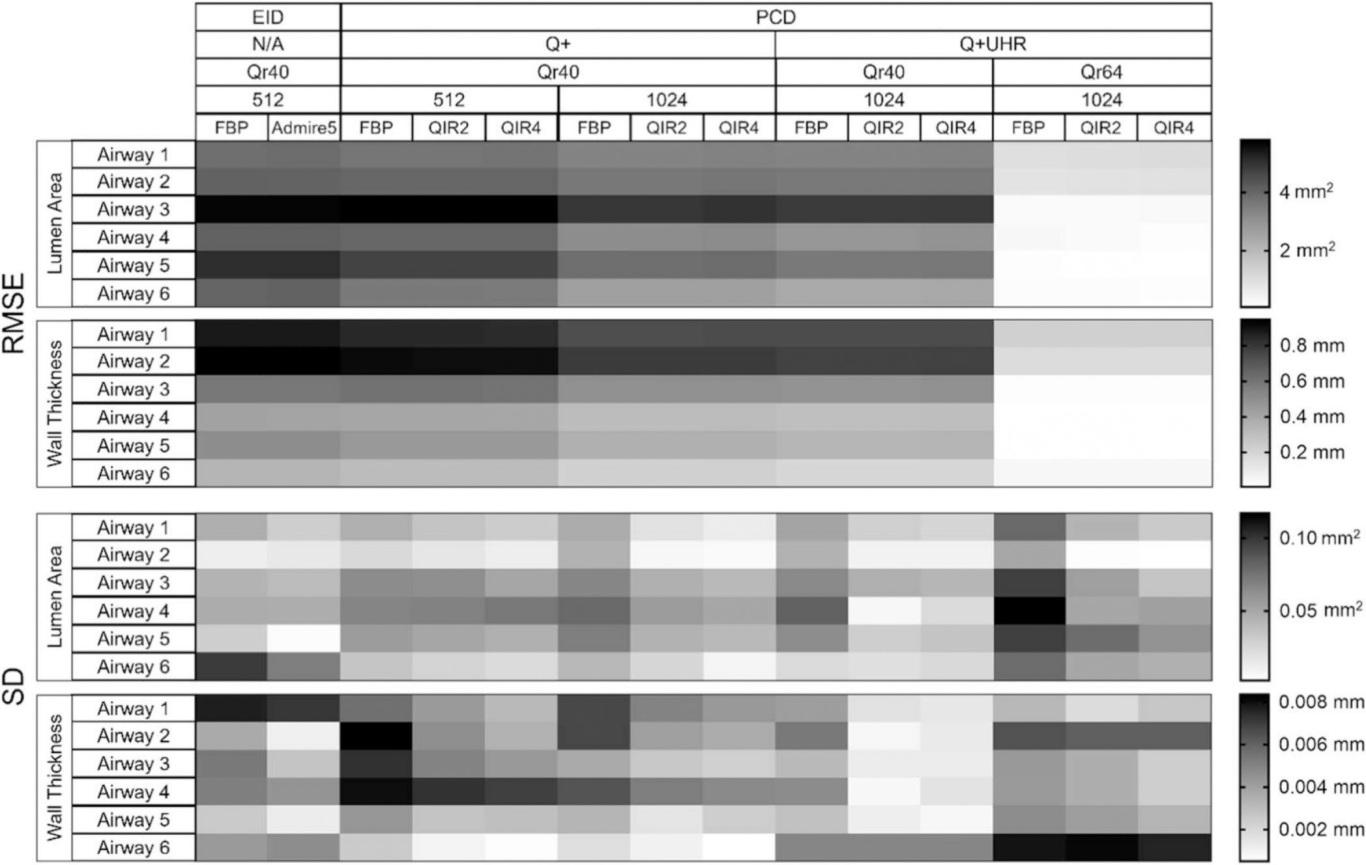
COPD lung phantom II airway analysis. Heatmap representations showing RMSE between mean measured and true airway dimensions across protocols (top) and SD of mean airway measurements across three repeated scans (bottom). Lower RMSE values (lighter color) indicate better agreement with reference standards. Lumen area and wall thickness measurements were generally consistent across reconstruction kernels. PCD protocols outperformed or matched EID-CT protocols in all cases with high repeatability, except for PCD Q+UHR with Qr64 and FBP reconstruction. Top: RMSE of Lumen Area and Wall Thickness was relatively similar across reconstruction kernels. PCD Q+UHR 1024 matrix with any reconstruction outperformed all other PCD protocols for all airways. across 3 repeated scans for EID and PCD protocols for Kyoto Phantom Lung Sample airways. PCD protocols outperformed or performed similarly to EID protocols for all airways. Bottom: The standard deviation of Lumen Area across three repeated scans was 0.10 mm^2^ or less for EID and PCD protocols, except for PCD Q+UHR Qr64 1024 matrix with FBP reconstruction kernel. The standard deviation of Wall Thickness across three repeated scans was less than 0.01 mm^2^ for all protocols and all airways. True Airway Measures: Airway 1 LA=7.1 mm^2^ and WT=0.6 mm, Airway 2 LA=7.1 mm^2^ and WT=0.6 mm, Airway 3 LA=28.3mm^2^ and WT=0.9mm, Airway 4 LA=28.3mm^2^ and WT=1.2 mm, Airway 5 LA=28.3 mm^2^ and WT=1.2 mm, Airway 6 LA=28.3 mm^2^ and WT= 1.5 mm.

Representative images of each airway in the COPD lung phantom for each protocol is shown in Figure 4. Lumen area (LA) and wall thickness (WT) for airway measures showed greater differences in RMSE across protocols (figure 5). PCD-CT protocols using the Q+UHR mode with the Qr64 kernel and a 1024 × 1024 matrix consistently showed better accuracy compared to other PCD and EID-CT protocols, with RMSE less than 2 mm^2^ for LA and 0.2 mm for WT (within 1 pixel). All PCD-CT protocols using the smoother Qr40 kernel had higher or equivalent RMSE values to EID-CT, irrespective of the mode and matrix, particularly for smaller airways. As the airway increased in size, the performance of PCD-CT protocols using the Qr40 kernel improved for all scan modes and matrix sizes. The effect of iterative reconstructions was negligible across protocols for both LA and WT measurements in the COPD *Lung Phantom II*.

The SD heatmap (bottom, figure 5) indicated minimal variability for LA measurements across repeated scans, with SD values below 0.10 mm^2^ for most protocols. Notably, PCD protocols using the Q+UHR mode with Qr64 kernel and FBP reconstruction exhibited slightly higher SD values, up to 0.10 mm^2^, across all iterative reconstructions, for LA measurements. However, the SD of the mean WT was markedly higher across the different protocols. The best performing repeatability as measured by the SD of the mean (remained below 0.01 mm for all airways) was the combination of Q+UHR with Qr40, 1024 × 1024 matrix, and QIR2 or 4 iterative reconstructions.

PCD-CT protocols with Q+UHR mode, Qr64 kernel, and 1024 matrix achieved the highest accuracy in airway measurements, while Q+UHR with Qr40 and iterative reconstruction offered the best repeatability, especially for wall thickness.

### 3.3. Kyoto phantom airways

**Figure 6.**
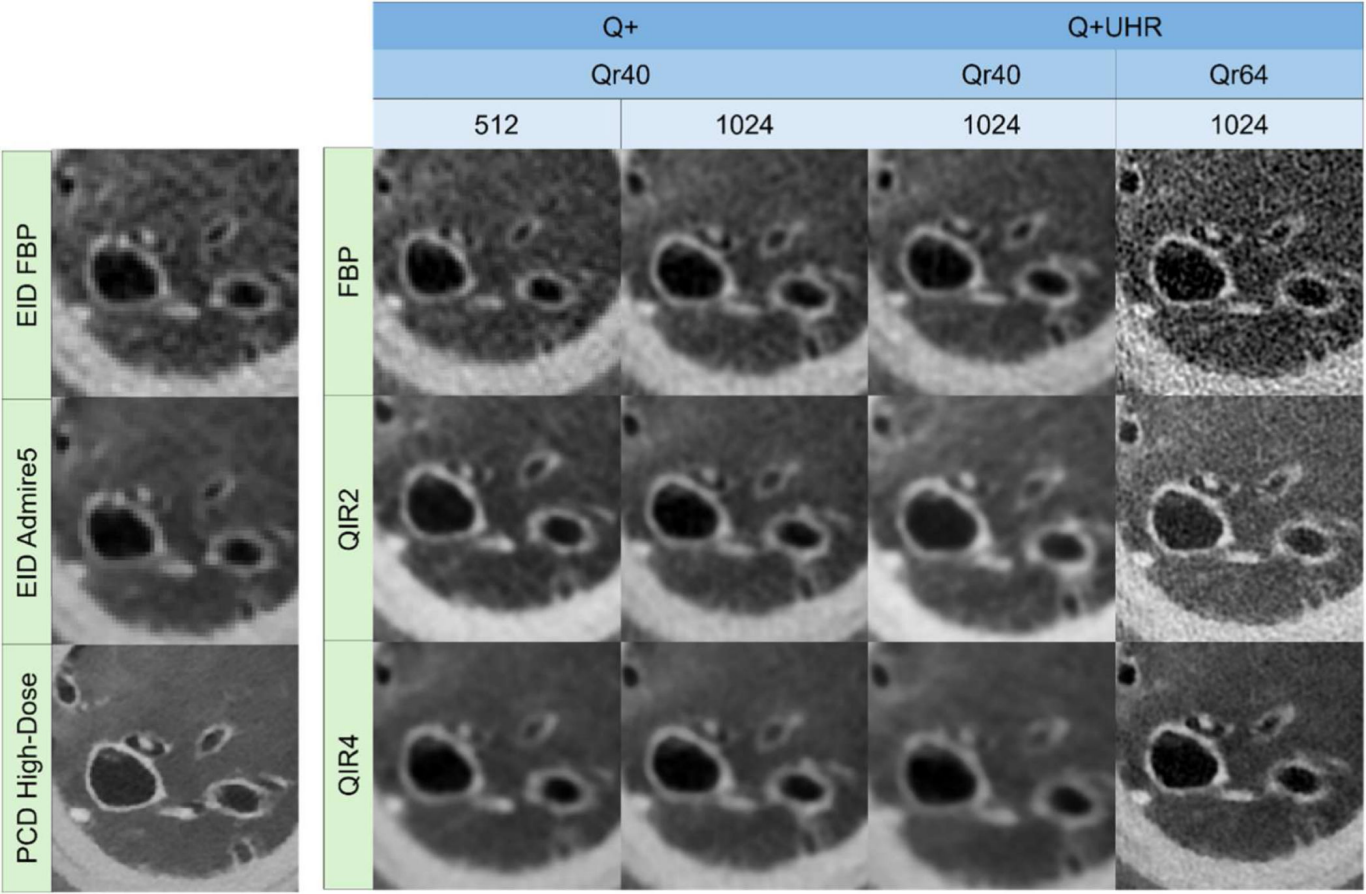
Representative images from Kyoto phantom for each protocol. Qualitative comparison of Kyoto ‘LUNGMAN’ phantom for EID FBP, EID Admire5 and PCD high-dose (left) and each low-dose PCD protocol (right).

**Figure 7.**
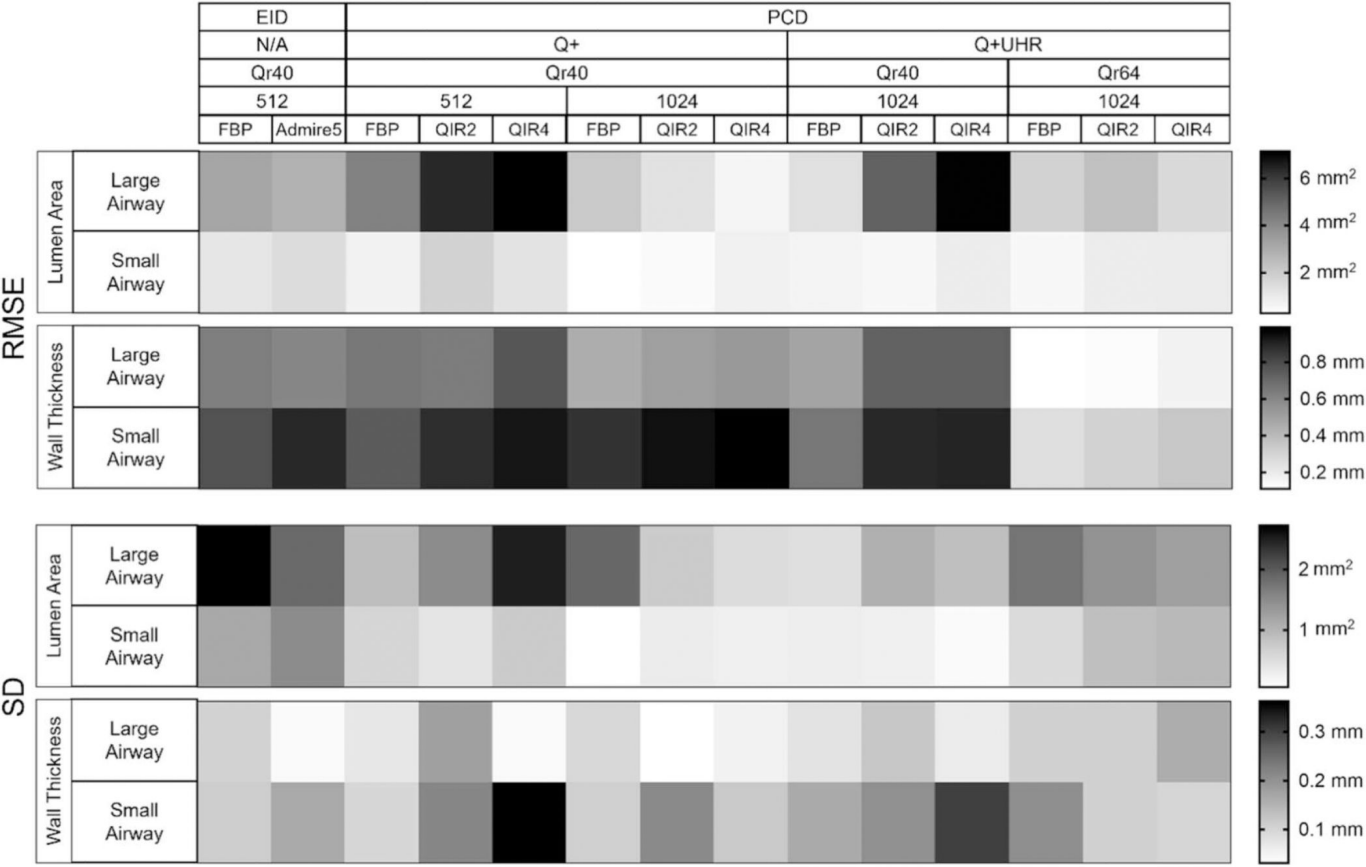
Kyoto phantom lung sample airway analysis. Heatmap representations showing RMSE between mean measured and ‘true’ airway dimensions across protocols (top) and SD of mean airway measurements across three repeated scans (bottom). Top: RMSE of Lumen Area across 3 repeated scans for EID and PCD protocols for Kyoto Phantom Lung Sample airways. For the Large Airway, 1024 matrix protocols had better performance than 512 matrix protocols overall, however QIR4 reconstruction RMSE was worse than FBP or QIR2 for Q+ Qr40 512 and Q+UHR Qr40 1024. Performance was relatively consistent across all protocols for Small Airway Lumen Area measurement. For Wall Thickness of both Large and Small Airway, Qr64 protocol outperformed all other protocols, which all had similar RMSE. Bottom: For Large Airway Lumen Area, the standard deviation was within approximately 2 mm^2^ for three repeated scans for all PCD protocols, except using PCD Q+ Qr40 512 matrix with QIR4 reconstruction. For Small Airway Lumen Area, the standard deviation was within 1.5 mm^2^ or less for all protocols. The standard deviation for Wall Thickness for both airways was 0.2 mm or less, except for PCD Q+ and Q+UHR Qr40 512 matrix with QIR4 reconstruction. *Note: The exact measurement of airway diameters of Lung Sample A airways is unknown. Therefore, standard measurements were estimated using a high-dose PCD-CT protocol (Mode: Q+UHR, Matrix: 1024 × 1024, CTDI_vol_: 12 mGy, Reconstruction Technique: FBP, Reconstruction Kernel: Qr64). ‘True’ Airway Measures: Large Airway LA=59.5 mm^2^ and WT=1.0 mm, Small Airway LA=5.3 mm^2^ and WT=0.8 mm.

Representative images of each airway in the Kyoto lung phantom for each protocol is shown in Figure 6. For large airway LA (true value = 59.5 mm^2^), PCD-CT protocols with a 1024 × 1024 matrix demonstrated superior accuracy to EID-CT (figure 7), with the lowest RMSE values observed for Q+ mode using the Qr40 kernel (RMSE<2mm^2^). Conversely, protocols using Q+ mode with a 512 × 512 matrix and displayed higher RMSE values (>6 mm^2^) to EID-CT, indicating reduced accuracy. LA measurements using FBP reconstruction were more representative of the true measure compared to QIR2/4 within the same PCD-CT protocol combination (figure 7).

For small airway LA (true value = 5.3 mm^2^), PCD-CT protocols were equivalent or slightly superior to EID-CT. Particularly using the Q+ mode with Qr40 kernel and 1024 × 1024 matrix and FBP, outperformed other protocols, achieving the lowest RMSE values across all reconstruction techniques (<1 mm^2^).

Equivalent to EID-CT, WT measurements for both large (true value = 1.0 mm) and small airways (true value = 0.8 mm) showed consistently high RMSE values across most PCD-CT protocols (RMSE >6 mm), with the Q+UHR mode with Qr64 kernel and 1024 × 1024 matrix protocol outperforming others (RMSE <0.4 mm). Airway measures were unaffected by the use of iterative reconstruction up to QIR4 across the measured ROIs except for the Q+UHR with Qr40 kernel and 1024 × 1024 matrix were FBP was the most accurate (figure 7).

For large airway LA, SD values remained within approximately 2 mm^2^ across all PCD-CT protocols (figure S2), except for PCD Q+Qr40 512 matrix combined with QIR4 reconstruction, which exhibited higher variability. For small airway LA, SD values were within 1.5 mm^2^ or less for all protocols. WT measurements for both airways showed SD values below 0.2 mm for most protocols.

Overall, PCD-CT protocols using Q+UHR mode and Qr64 kernel demonstrated superior accuracy and consistency in measuring both LA and WT for large and small airways.

### 3.4. Spatial resolution

The spatial resolution of PCD-CT protocols was assessed using MTF curves, with MTF (%) plotted against spatial frequency (cycles/cm) for each protocol using Kyoto (figure S1). For protocols using a 512 × 512 matrix, the MTF cutoff was similar across reconstruction algorithms (FBP, QIR2, and QIR4) and remained comparable between PCD-CT Q+ and EID-CT protocols indicating that sampling was limiting. However, the MTF cutoff for protocols using a 1024 × 1024 matrix, PCD-CT in Q+UHR mode demonstrated a marked improvement in spatial resolution, achieving higher values at higher spatial frequencies compared to other protocols. Q+UHR with the Qr64 kernel consistently showed the highest cutoff value for the MTF with nominal spatial resolution closer to the Nyquist frequency of 14.027 cycles/cm.

In summary, PCD-CT protocols using Q+UHR mode with a 1024 matrix and Qr64 kernel achieved the highest spatial resolution, approaching the Nyquist limit, while 512 matrix protocols were resolution-limited by sampling.

### 3.5. Noise performance: Signal-to-Noise Ratio (SNR) and Contrast-to-Noise Ratio (CNR)

The SNR and CNR performance are highly dependent on the kernel, the matrix size, and the use of iterative reconstruction strength as expected. Among PCD-CT protocols, the QIR4 reconstruction enhanced both SNR and CNR compared to FBP and QIR2 reconstructions. PCD-CT protocols using Qr40 kernel and QIR4 demonstrated a notable improvement in SNR, and outperformed EID-CT. PCD-CT protocols using Q+UHR mode with the Qr64 kernel and 1024 × 1024 matrix showed the lowest SNR values due to higher noise power in combination with smaller pixel size. For CNR, a similar trend was observed. For equivalent kernel, matrix size, and comparable iterative reconstruction, the CNR performance was superior in PCD-CT as expected given the underlying physical advantages of the PCD. Not surprisingly, PCD-CT in Q+UHR mode with the Qr64 kernel had lower CNR than all other protocols as shown in figure S2, but this tradeoff may be countered by the superior spatial resolution when using QIR4 reconstruction, which is comparable to the SNR and CNR performance of the EID-CT with FBP recon.

## 4. Discussion

PCD-CT detector design allows for improved image quality, sharpness, and resolution over EID-CT without increasing radiation dose. In this study, we evaluated the performance of low-dose (~2.2 mGy CTDI_vol_) PCD-CT protocols for measuring density and airway morphometry, relative to the conventional low-dose EID-CT protocols widely used for longitudinal studies of obstructive lung disease [14–16]. Overall, this study demonstrated that PCD-CT protocols, particularly those using the Q+UHR mode with 1024 × 1024 matrix, consistently achieved the highest accuracy and repeatability in airway measurements across phantoms, but with differing reconstruction kernel for a given quantitative task. For lung-mimicking foam densities, PCD-CT with Qr40 kernel outperformed or matched EID-CT protocols, with markedly improved RMSE values except in tracheal air, where variability was higher in high-resolution modes. The use of the Qr64 kernel resulted in unacceptably high variability in density measures (e.g., tracheal air measurements SD up to ±9 HU for PCD-CT). Conversely, airway lumen area (LA) and wall thickness (WT) measurements were most accurate using Q+UHR with Qr64, while Q+ protocols with Qr40 and 1024 matrix offered excellent performance for LA with minimal variability. MTF analysis confirmed superior spatial resolution with Q+UHR protocols and 1024 matrix, approaching the Nyquist frequency. Additionally, QIR4 iterative reconstruction significantly improved SNR and CNR for PCD-CT, especially when combined with Qr40, outperforming EID-CT using ADMIRE5 (figure S2). Despite trade-offs in noise and contrast with high-resolution protocols, PCD-CT consistently demonstrated superior or comparable performance to EID-CT across all quantitative measures evaluated.

The impact of these results on quantitative trials is substantial as this CT technology becomes more commonly used in the clinic and research arenas. First, our results support the integration of PCD-CT systems into clinical research with best-matched settings corresponding to the Q+, Qr40, and QIR 4 settings. Either a 512 or 1024 matrix can be used but a 1024 matrix has obvious benefits from a pure sampling resolution perspective. Measures were highly repeatable as well with the difference in density measures within 2 HU for most measurements and most protocols in agreement with the SD of the same density foams for standardized EID-CT protocol development [30]. Second, the Q+UHR acquisition mode offers a substantial improvement in airway measures given access to larger reconstruction matrix sizes. In this mode with the higher resolution quantitative Qr64 kernel, RMSE for the smallest airways (3 mm inner diameter) were within 0.2 mm and even smaller errors were observed for larger (≥ 6 mm inner diameter) airways compared to more than 0.6 mm RMSE for EID-CT of the same airways. Moreover, the MTF findings highlight the significant advantage of Q+UHR mode with the Qr64 kernel in achieving superior spatial resolution, particularly for high-frequency details, making it the optimal choice for protocols requiring fine spatial detail.

While choice of the Q+UHR mode decreased repeatability slightly, the standard deviations for the airway measures were within 1% of the mean value, or less than the size of a single voxel for all airways in all cases. A reasonable option, therefore, would be to use the Q+UHR mode and to optimize the reconstruction separately for density using the smooth Qr40 kernel and for airway measures using Qr64 kernel with the higher frequency cut-off. In this way, an accuracy for parenchymal density and airways could be achieved, while still matching performance to that of EIC-CT systems if desired (i.e., using the Qr40 reconstruction for both density and airways measures).

SNR and CNR values for the PCD versus EID results were as expected given the theoretical advantage of PCD with respect to electronic noise and reduced streak artifact because of the virtual monoenergetic reconstruction [31]. Nonetheless, many other studies have found more dramatic improvements in SNR and CNR for PCD systems [8, 13, 32]. In this study, we quantitatively compared performance for matched relatively low doses and parameters in the same imaging phantom for quantitative protocols and found that SNR and CNR were not dramatically improved for PCD versus EID-CT when comparing equivalent kernel, matrix size, and FBP reconstruction. Because the dose here using CTDI_vol_ is low and matched to the extent possible between the EID and PCD systems, non-linear filtering may play a role in further reducing noise for EID-CT, even for FBP reconstruction [33], artificially increasing SNR and CNR performance for EID-CT. Otherwise, the quantitative ratios trend with expected improvement of noise performance with kernel, the QIR2 to QIR4 iterative reconstructions, and matrix size.

As noted, the PCD results point to likely improvements in the accuracy and consistency of density measures in the range of air and lung parenchyma. RMSE values were consistently lower for PCD compared to EID across all densities, particularly for air and the low density NIST foam standards when reconstructed using the Q+ mode and smooth Qr40 kernel. These results confirm that Qr40-based PCD-CT protocols provide the most accurate parenchymal density measurements among all tested conditions.

Interestingly, RMSE became less accurate for the higher density 20 lb. foam for all acquisition protocols, suggesting errors may increase for higher density materials. The Q+UHR with the Qr64 kernel was comparatively less accurate, although RMSE was comparable to that for the EID CT using Qr40. The Q+UHR Qr64 and EID CT struggled with the accuracy and repeatability of tracheal air. This likely stems from noise and scatter, respectively. The use of Q+ and Q+UHR with Qr40 largely eliminated these errors and improved repeatability.

While Q+UHR with Qr64 demonstrated the most accurate airway morphometry, performance varied between the COPD Phantom II ‘airways’ (i.e., acrylic tubes) and the anatomic airways preserved within the Kyoto phantom. Two differences in these measurements might explain the differences in performance. First, the airway geometry is more complex, and dimensions vary with axial slice number. Given that we are using a semi-quantitative measurement method in the Kyoto airways on a single slice, there is the potential for variation in measurement due to seed position and only a single reader conducted this semi-automated analysis, a source of potential bias. Moreover, there were likely small variations in the slice location between scans with respect to the airway structure that could alter the axial slice selected for analysis.

Dose reduction is a key priority in clinical CT imaging, particularly for longitudinal studies in large multi-center research trials and in lung cancer screening and interstitial lung disease, who often undergo repeated scans for monitoring and follow-up. This pre-clinical study offers rigorous treatment of parameter settings for both acquisition and reconstruction to facilitate robust emphysema and airway measures at low CT dose using PCD-CT, a balance that requires tradeoffs in SNR and CNR with spatial resolution. This study showed that this balance can be achieved with a single acquisition with PCD-CT in Q+UHR mode with 1024 matrix size and QIR2 and Qr40 reconstructions to balance noise for improved density measures, and QIR4 and Qr64 in a high-resolution reconstruction to mitigate increased noise while achieving higher resolution for airway measures. Our findings are consistent with emerging clinical evidence highlighting the advantages of PCD-CT in pulmonary imaging at low CT dose. Multiple studies have shown that PCD-CT enables a 43%–50% reduction in radiation dose compared to EID-CT, while maintaining comparable image quality [8, 32]. Although some studies [8, 34] reported slightly lower SNR with PCD-CT at reduced dose levels, this was attributed to higher spatial resolution and sharper reconstruction kernels and did not affect subjective diagnostic confidence. Additionally, ultra-high-resolution (UHR) PCD-CT imaging (0.2 mm) demonstrated superior visualization of fine bronchial structures, vessels, and nodules [35]. In ILD studies [36], UHR-PCCT improved reader confidence in detecting subtle parenchymal changes and enabled significant dose reduction (up to 66%) without compromising diagnostic performance. Ultimately, the integration of PCD-CT into quantitative lung CT for longitudinal studies could improve quantitative measures while reducing inter-scanner variability, both of which would increase sensitivity to early or subtle disease changes for both research and routine care.

Several limitations should be considered in interpreting these findings. First, for the reference standard airway measurements in the Kyoto phantom, we used a high-dose (~9 mGy CTDI_vol_) PCD-CT scan in Q+UHR mode with Qr64 kernel and 1024 matrix. We acknowledge that this approach introduces a degree of circularity, since the same scanner and reconstruction settings were also evaluated for the low-dose scans. This was necessary due to the lack of true ground truth measurements for these anatomical airways. Second, anisotropy in spatial resolution, specifically, higher in-plane than through-plane resolution, may limit the accuracy of 3D morphometric measures. At the 2.2 mGy CTDI_vol_ used in this study, slice thickness could not be reduced without introducing unacceptable noise. As such, anisotropic resolution was a trade-off to achieve superior in-plane accuracy using Q+UHR mode. Lastly, we focused on a limited protocol space, to reflect clinically relevant conditions used in the COPDGene EID-CT Siemens Force system [20] protocol which was presumed to bracket the performance for the same scanner manufacturer of the Naeotom Alpha PCD-CT system. While this increases comparability for low dose performance that would be realistically implemented for a longitudinal trial like COPD Gene and SPIROMICS, it may not reflect the full range of PCD-CT capabilities.

## 5. Conclusion

Low-dose PCD-CT protocols demonstrate superior density accuracy and spatial resolution compared to EID-CT, particularly for airway measures when using the Q+UHR mode with the Qr64 kernel and a 1024 × 1024 matrix. These configurations provide accurate density measures in lung equivalent foams with improved airway measurements in airway mimicking tubes and preserved lung tissue, making PCD-CT a promising modality for quantitative lung imaging.

Among PCD-CT protocols at the low 2.2 mGy CTDI_vol_, Q+UHR mode with Qr40 kernel and QIR4 reconstruction offered an optimal balance of spatial resolution and image quality, outperforming EID-CT in SNR and CNR. These findings support the potential of PCD-CT as a superior alternative to EID-CT for preclinical lung imaging at low radiation doses.

## Supplementary Material

supplementary materials

Supplementary material for this article is available online

## Figures and Tables

**Table 1.** CT scanning protocols for EID and PCD.

	EID	PCD	

Scanner	SOMATOM Force	NAEOTOM Alpha	
Mode	—	Q+	Q+UHR
FOV (mm^2^)	260 × 260	260 × 260	260 × 260
Matrix	512 × 512	512 × 512 and 1024 × 1024	1024 × 1024
Pitch	1.0	1.0	1.0
Detector Config	192 × 0.6 mm	144 × 0.4 mm	120 × 0.2 mm
Rotation Time (s)	0.25	0.25	0.25
Tube voltage (kV)	120	120	120
CARE Dose	4D (QRmAs = 25)	On (KeVIQ= 19)	On (KeVIQ= 19)
CTDI_vol_ (mGy)	2.24	2.22	2.27
DLP (mGy*cm)	67.2	66.6	68.1
Slice Thickness (mm)	0.75	0.8	0.8
Pixel Spacing (mm)	0.5	0.5	0.5
Virtual ME Recon	—	70keV	70keV
ReconTechnique	FBP,ADMIRE5	FBP, QIR2, QIR4	FBP, QIR2, QIR4
Recon Kernel	Qr40	Qr40	Qr40Qr64

Abbreviations: CT=computed tomography, CTDI_vol_ =volume CT dose index, DLP=dose length product, EID=energy integrating detector, FOV=field of view, IR=Iterative reconstruction, PCD=photon-counting detector, UHR=Ultra high resolution, ADMIRE5=Advanced Modeled Iterative Reconstruction with relative weighting value of 5. ADMIRE is the model based iterative reconstruction on the Force EID CT, QIR=quantum iterative reconstruction is the model-based iterative reconstruction on the Naeotom photon-counting detector CT. QIR4 is the greatest relative weighting of the model-based reconstruction setting available.

**Table 2.** Reference standard measurements for RMSE calculations.

Parameter	Standard measurement

*Kyoto Phantom*	
Density (HU)	
NIST4	−939
NIST12	−822
NIST20	−697
Water	0
Air—Inside lung	−1000
Air—Trachea	−1000
Large Airway^a^	
LA (mm^2^)	59.5
WT (mm)	1.0
Small Airway^a^	
LA (mm^2^)	5.3
WT (mm)	0.8

*COPD Lung Phantom II*	

Airway1	
LA (mm^2^)	7.1
WT (mm)	0.6
Airway2	
LA (mm^2^)	7.1
WT (mm)	0.6
Airway3	
LA (mm^2^)	28.3
WT (mm)	0.9
Airway4	
LA (mm^2^)	28.3
WT (mm)	1.2
Airway5	
LA (mm^2^)	28.3
WT (mm)	1.2
Airway6	
LA (mm^2^)	28.3
WT (mm)	1.5

a The exact measurement of airway diameters of Lung Sample A airways is unknown. Therefore, standard measurements were estimated using a high-dose PCD-CT protocol (Mode: Q +UHR, Matrix: 1024 × 1024, CTDI_vol_: 12 mGy, Reconstruction Technique: FBP, Reconstruction Kernel: Qr64).

## Data Availability

All data that support the findings of this study are included within the article (and any supplementary files). Data will be available from 30 October 2025.
