# Supplementary material for: Comparison of quantitative lung measures in low dose energy-integrating detector and photon-counting detector chest CT with an anthropomorphic phantom
Source: Biomed Phys Eng Express. Author manuscript; Available in PMC 2026 Jun 30. (PMC13317018; doi:10.1088/2057-1976/ae0e27)
Supplement: supplementary materials [file NIHMS2169155-supplement-supplementary_materials.pdf]

Comparison of Quantitative Lung Measures in Low Dose Energy-Integrating Detector and Photon-Counting Detector Chest CT with an Anthropomorphic Phantom

Supplementary Materials

Table S1. Quantitative measures of noise, density, and airway dimensions

	Reference	EID		PCD											
Mode	-	-	-	Q+						Q+UHR					
Recon Kernel	-	Qr40	Qr40	Qr40						Qr40			Qr64		
Matrix Size	-	512×512	512×512	512×512			1024×1024			1024×1024			1024×1024		
Recon Technique	-	FBP	Admire5	FBP	QIR2	QIR4	FBP	QIR2	QIR4	FBP	QIR2	QIR4	FBP	QIR2	QIR4
	Kyoto Phantom														
Density (HU)															
NIST4	-939	-931±2	-931±1	-942±1	-943±1	-943±1	-941±1	-941±1	-942±0	-941±2	-943±1	-943±1	-950±2	-950±2	-950±2
NIST12	-822	-819±1	-819±1	-820±1	-821±0	-820±1	-820±1	-820±1	-820±1	-822±1	-823±0	-823±0	-830±1	-831±2	-831±2
NIST20	-681	-681±1	-681±1	-686±2	-685±2	-686±1	-686±2	-686±2	-686±1	-686±1	-686±1	-686±1	-689±1	-689±1	-689±1
Water	0	1±2	1±2	0±1	0±1	0±1	2±0	1±0	1±1	-1±3	1±1	1±1	-1±3	-2±3	-2±3
Air – Inside lung	-1000	-990±0	-1001±1	-1001±2	-1001±1	-1001±1	-1002±2	-1001±1	-1001±1	-1002±3	-999±1	-999±1	-1016±2	-1016±1	-1016±1
Air – Trachea	-1000	-973±1	-980±0	-997±1	-998±1	-999±1	-995±0	-996±0	-997±0	-994±8	-1004±1	-1005±1	-1007±9	-1004±8	-1003±9
Lung parenchyma	-	-589±0	-588±0	597±0	-595±1	-596±0	-592±0	-591±0	-590±0	-593±3	-592±2	-592±2	-605±2	-602±2	-597±2
Large Airway															
LA (mm^2)	59.5*	57.9±3.3	57.4±2.3	55.4±1.1	53.3±1.8	52.9±3	60.5±2.3	60.6±0.9	59.2±0.6	58.3±0.6	54.6±1.3	52.5±1.1	60.4±2.7	61.4±1.7	60.5±1.6
WT (mm)	1.0*	1.6±0.1	1.6±0.0	1.7±0.1	1.6±0.2	1.8±0.0	1.5±0.1	1.5±0.0	1.6±0.1	1.5±0.1	1.7±0.2	1.8±0.1	1.0±0.2	1.0±0.1	1±0.2
Small Airway															
LA (mm^2)	5.3*	5.5±1.4	5.2±1.8	4.9±0.8	3.6±0.5	4.4±0.9	5.1±0.1	5.0±0.4	4.6±0.3	4.7±0.3	4.9±0.3	4.4±0.1	5.4±0.8	5.0±1.1	5.1±1.2
WT (mm)	0.8*	1.5±0.1	1.6±0.2	1.5±0.1	1.6±0.3	1.6±0.4	1.6±0.1	1.7±0.3	1.7±0.1	1.4±0.2	1.6±0.2	1.6±0.4	1.0±0.3	1.1±0.1	1.1±0.1
	COPD Lung Phantom II														
Airway 1															
LA (mm^2)	7.1	3.3±0.0	3.2±0.0	3.4±0.1	3.4±0.0	3.4±0.0	3.8±0.0	3.7±0.0	3.7±0.0	3.8±0.1	3.7±0.0	3.7±0.0	6.1±0.1	6.0±0.1	6.0±0.0
WT (mm)	0.6	1.5±0.0	1.5±0.0	1.4±0.0	1.4±0.0	1.4±0.0	1.3±0.0	1.3±0.0	1.3±0.0	1.3±0.0	1.3±0.0	1.3±0.0	0.8±0.0	0.8±0.0	0.8±0.0
Airway 2															
LA (mm^2)	7.1	3.0±0.0	3.0±0.0	3.1±0.0	3.1±0.0	3.1±0.0	3.5±0.0	3.4±0.0	3.4±0.0	3.6±0.0	3.5±0.0	3.4±0.0	6.2±0.0	6.2±0.0	6.1±0.0
WT (mm)	0.6	1.5±0.0	1.5±0.0	1.5±0.0	1.5±0.0	1.5±0.0	1.4±0.0	1.4±0.0	1.4±0.0	1.4±0.0	1.4±0.0	1.4±0.0	0.8±0.0	0.8±0.0	0.8±0.0
Airway 3															
LA (mm^2)	28.3	22.6±0.1	22.6±0.0	22.4±0.1	22.4±0.1	22.5±0.1	23.4±0.1	23.3±0.1	23.3±0.1	23.5±0.1	23.4±0.1	23.4±0.1	28.1±0.1	28.1±0.1	28±0.0
WT (mm)	0.9	1.5±0.0	1.5±0.0	1.5±0.0	1.5±0.0	1.5±0.0	1.4±0.0	1.4±0.0	1.4±0.0	1.4±0.0	1.4±0.0	1.4±0.0	0.9±0.0	0.9±0.0	0.9±0.0
Airway 4															
LA (mm^2)	28.3	24.2±0	24.2±0.1	24.3±0.1	24.3±0.1	24.3±0.1	25.2±0.1	25.2±0.1	25.1±0.1	25.5±0.0	25.4±0.0	25.3±0.0	28.6±0.1	28.5±0.1	28.4±0.1
WT (mm)	1.2	1.6±0.0	1.6±0.0	1.6±0.0	1.6±0.0	1.6±0.0	1.5±0.0	1.5±0.0	1.5±0.0	1.5±0.0	1.5±0.0	1.5±0.0	1.2±0.0	1.2±0.0	1.2±0.0
Airway 5															
LA (mm^2)	28.3	23.2±0.0	23.2±0.0	23.6±0.1	23.6±0.1	23.6±0.1	24.5±0.1	24.4±0.1	24.4±0.0	24.8±0.0	24.7±0.0	24.6±0.0	28.3±0.1	28.3±0.1	28.2±0.1
WT (mm)	1.2	1.7±0.0	1.7±0.0	1.7±0.0	1.7±0.0	1.7±0.0	1.6±0.0	1.6±0.0	1.6±0.0	1.5±0.0	1.5±0.0	1.5±0.0	1.2±0.0	1.2±0.0	1.2±0.0

Airway 6															
LA (mm^2)	28.3	24.3±0.1	24.2±0.1	24.7±0.0	24.7±0.0	24.7±0.0	25.5±0.1	25.6±0.0	25.6±0.0	25.8±0.0	25.8±0.0	25.8±0.0	28.4±0.1	28.4±0.0	28.3±0.1
WT (mm)	1.5	1.8±0.0	1.8±0.0	1.8±0.0	1.8±0.0	1.8±0.0	1.7±0.0	1.7±0.0	1.7±0.0	1.7±0.0	1.7±0.0	1.7±0.0	1.5±0.0	1.5±0.0	1.5±0.0

Data is reported as mean±standard deviation. * Measures obtained using a high-dose (8.93 mGy $CTDI_{vol}$) PCD-CT reference acquisition (Q+UHR, 1024×1024 matrix, Qr64 kernel). Abbreviations: EID=energy integrating detector; PCD=photon-counting detector; LA=lumen area; WT=wall thickness

Table S2. Quantitative measures of noise, density, and airway deviation from their standard reference by root mean squared error (RMSE)

	EID		PCD												
Mode	-	-	Q+						Q+UHR					
Recon Kernel	Qr40	Qr40	Qr40						Qr40			Qr64		
Matrix Size	512×512	512×512	512×512			1024×1024			1024×1024			1024×1024		
Recon Technique	FBP	Admire5	FBP	QIR2	QIR4	FBP	QIR2	QIR4	FBP	QIR2	QIR4	FBP	QIR2	QIR4
Kyoto Phantom														
Density (HU)														
NIST4	7.77	7.65	3.77	4.33	4.19	2.67	2.80	2.90	2.56	4.49	4.70	10.29	11.44	11.27
NIST12	3.45	3.48	2.31	1.34	1.74	1.78	1.76	1.76	0.76	0.84	1.00	6.67	8.94	8.86
NIST20	16.27	16.35	11.38	12.11	10.99	11.11	11.19	11.25	10.95	11.47	11.23	9.20	7.99	8.04
Water	1.96	1.84	1.18	0.87	1.20	1.70	1.52	1.39	2.60	1.28	1.36	2.03	2.63	2.63
Air – Inside lung	9.77	1.57	1.57	1.53	1.34	2.04	1.75	1.58	2.84	1.12	1.11	12.83	15.85	15.92
Air – Trachea	27.11	20.15	2.91	2.41	1.12	5.06	3.70	2.83	9.10	4.24	4.97	10.05	7.87	8.11
Large Airway														
LA (mm^2)	3.20	2.85	4.26	6.37	7.13	2.11	1.28	0.62	1.35	5.08	7.06	1.80	2.37	1.57
WT (mm)	0.62	0.59	0.65	0.63	0.76	0.45	0.51	0.53	0.49	0.72	0.73	0.11	0.12	0.16
Small Airway														
LA (mm^2)	1.16	1.49	0.73	1.81	1.23	0.29	0.42	0.78	0.69	0.51	0.93	0.52	0.92	0.96
WT (mm)	0.77	0.89	0.74	0.87	0.93	0.86	0.94	0.98	0.65	0.89	0.89	0.25	0.30	0.34
COPD Lung Phantom II														
Airway 1														
LA (mm^2)	3.82	3.86	3.66	3.66	3.69	3.32	3.33	3.37	3.34	3.34	3.38	1.01	1.02	1.09
WT (mm)	0.88	0.88	0.83	0.83	0.83	0.73	0.73	0.73	0.73	0.73	0.73	0.22	0.22	0.22
Airway 2														
LA (mm^2)	4.07	4.07	3.97	3.97	3.99	3.61	3.62	3.66	3.58	3.58	3.62	0.87	0.88	0.93
WT (mm)	0.94	0.94	0.90	0.90	0.90	0.78	0.78	0.79	0.76	0.76	0.77	0.17	0.17	0.17
Airway 3														
LA (mm^2)	5.66	5.67	5.81	5.81	5.80	4.95	4.95	5.00	4.85	4.85	4.89	0.20	0.21	0.26
WT (mm)	0.59	0.58	0.60	0.60	0.60	0.49	0.49	0.49	0.48	0.48	0.48	0.01	0.01	0.01
Airway 4														
LA (mm^2)	4.08	4.10	3.99	4.00	4.00	3.09	3.10	3.15	2.89	2.89	2.97	0.26	0.19	0.12
WT (mm)	0.41	0.41	0.40	0.40	0.40	0.30	0.31	0.31	0.29	0.29	0.30	0.006	0.007	0.007
Airway 5														
LA (mm^2)	5.07	5.10	4.68	4.68	4.68	3.81	3.82	3.87	3.58	3.59	3.63	0.09	0.08	0.08
WT (mm)	0.50	0.50	0.46	0.46	0.45	0.36	0.36	0.36	0.33	0.34	0.34	0.005	0.006	0.005
Airway 6														
LA (mm^2)	4.03	4.09	3.57	3.57	3.55	2.68	2.68	2.65	2.44	2.43	2.44	0.16	0.13	0.09
WT (mm)	0.34	0.34	0.31	0.31	0.31	0.21	0.21	0.21	0.19	0.19	0.19	0.03	0.03	0.03

Data is reported as Root Mean Squared Error (RMSE). Abbreviations: EID=energy integrating detector; PCD=photon-counting detector; LA=lumen area; WT=wall thickness

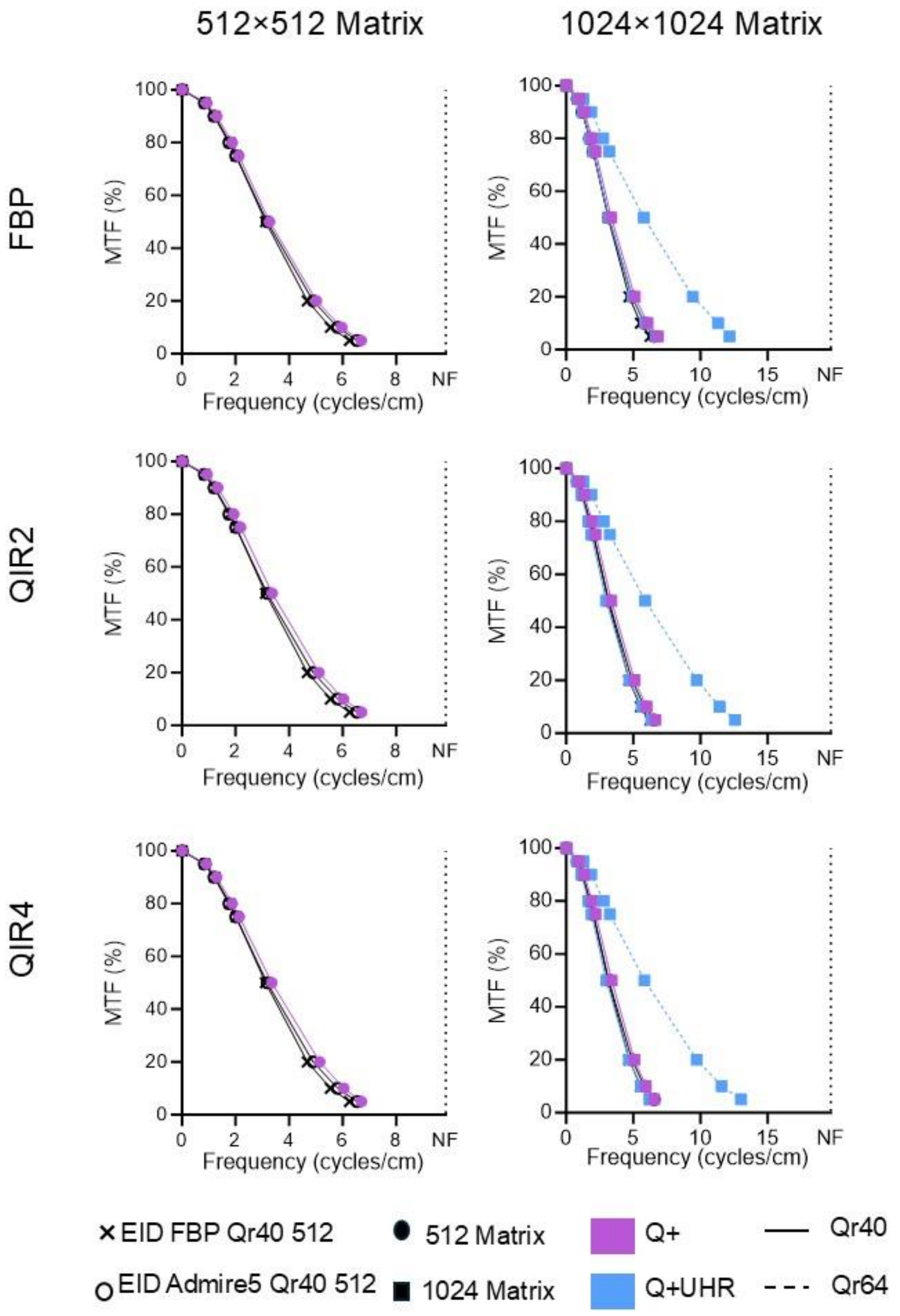

Figure S1. MTF for Kyoto

Modulation transfer function (%) plotted against the frequency for each protocol. The Nyquist Frequency (NF) for PCD is shown on plots as dotted vertical lines (512 matrices=9.846; 1024 matrices=19.692). MTF was similar across reconstruction algorithms (FBP, QIR2, QIR4). For 512 matrices (left), MTF was similar for EID and Q+ Qr40. For 1024 matrices, the MTF for Q+ Qr40 and Q+UHR Qr40 were similar to MTF for EID; Q+UHR Qr64 MTF outperformed all other protocols. ADMIRE5=Advanced Modeled Iterative Reconstruction with relative weighting value

of 5. ADMIRE is the model based iterative reconstruction on the Force EID CT, QIR=quantum iterative reconstruction is the model-based iterative reconstruction on the Naeotom photon-counting detector CT. QIR4 is the greatest relative weighting of the model-based reconstruction setting available.

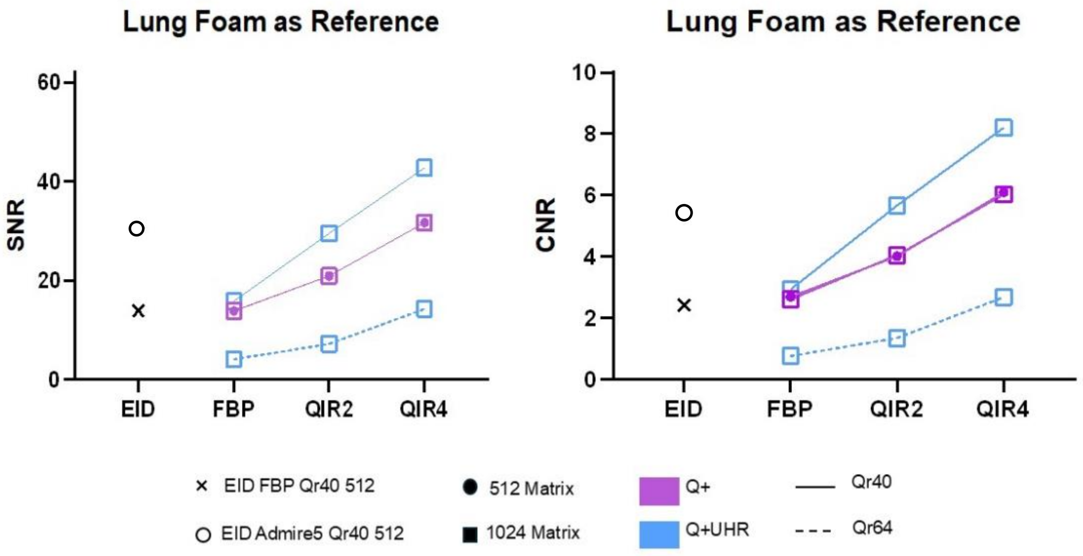

Figure S2. Signal-to-noise (SNR) and contrast-to-noise (CNR) ratios measurements in the lungs for Kyoto Phantom. FBP – "Filtered Back Projection;" EID – "Energy Integrating Detector" using the reference protocol. ADMIRE5=Advanced Modeled Iterative Reconstruction with relative weighting value of 5. ADMIRE is the model based iterative reconstruction on the Force EID CT, QIR=quantum iterative reconstruction is the model-based iterative reconstruction on the Naeotom photon-counting detector CT. QIR4 is the greatest relative weighting of the model-based reconstruction setting available.

SNR was calculated using the mean density measured in the lung equivalent foam divided by a noise estimate from the standard deviation of the density measured within an identical ROI placed in the lung equivalent foam from a difference image calculated from two repeated scans of the same protocol.

CNR was calculated as the difference in mean density measured between the NIST12 foam and NIST8 foam within the same scan, divided by a noise estimate, identical to that used for SNR, i.e. the standard deviation of the density measured within an ROI placed in the lung equivalent foam from a difference image calculated from two repeated scans of the same protocol.
